# Late Pancreatic Fistula After Pancreaticoduodenectomy: A Case Report and Review of the Literature

**DOI:** 10.1089/crpc.2016.0015

**Published:** 2016-11-01

**Authors:** Numa P. Perez, David G. Forcione, Cristina R. Ferrone

**Affiliations:** ^1^ Department of Surgery, Massachusetts General Hospital, Boston, Massachusetts.; ^2^ Department of Gastroenterology, Massachusetts General Hospital, Boston, Massachusetts.

**Keywords:** ampullary adenocarcinoma, pancreatic fistula, pancreaticojejunostomy stricture

## Abstract

**Background:** More than 100 years after its conception, the pancreaticoduodenectomy (PD) remains a challenging procedure with significant morbidity, often due to a postoperative pancreatic fistula (POPF). Factors related to patient physiology, tumor anatomy/pathology, and surgeon/surgical technique have been studied, yielding results at times conflicting and difficult to reproduce. We present a case of a late POPF along with a brief review of the current literature.

**Case Presentation:** The patient is a 55-year-old female with a 20 pack-year smoking history and no history of alcohol abuse, who presented for evaluation of new nausea. Her laboratory tests and computed tomography (CT) imaging were suggestive of biliary obstruction. She was found to have an invasive ampullary adenocarcinoma and subsequently underwent a classic PD. She developed a POPF, managed through a closed suction drain placed intraoperatively. Her course was complicated by the development of an intra-abdominal abscess, managed percutaneously through CT-guided placement of two drains, subsequently removed without issues. She recovered uneventfully until 8 months after the operation, when she presented with abdominal pain and pancreatitis. She was found to have an intra-abdominal collection, again managed percutaneously via CT-guided drainage. This time, the amylase and lipase levels of the drainage fluid were 21,860 and 86,650 U/L, respectively, and cultures were sterile. Upon workup of her pancreatic fistula, a severe stricture at the pancreaticojejunostomy (PJ) was identified. She underwent endoscopic placement of a Hobbs stent by the GI service.

**Conclusion:** Although commonly diagnosed in the days to weeks after a PD, we present a case of a POPF that manifested 8 months after the initial operation in association with a PJ stricture. This case highlights the importance of considering the diagnosis even months after the operation in a patient who presents with symptoms of pancreatitis and/or imaging findings consistent with an intra-abdominal collection.

## Introduction and Background

More than 100 years after its conception by Dr. Walther Kausch, and more than 80 years after Dr. Allen Oldfather Whipple's description of his revised version, the pancreaticoduodenectomy (PD) continues to be a difficult operation for both surgeons and patients alike. The pancreaticoenteric anastomosis is often challenging and is commonly the source of postoperative complications. The first obstacle to studying postoperative pancreatic fistulas (POPFs) was the lack of a universally accepted definition. In 2004, the International Study Group on Pancreatic Fistula (ISGPF) was formed, which consisted of 37 pancreatic surgeons from 15 countries, and had the purpose of standardizing a definition that could be used thereon to study the phenomenon. The results of such a group effort were presented in a landmark publication in 2005^1^ (Table 1).

**Table 1.** **Definition and Grading of Postoperative Pancreatic Fistula According to the International Study Group on Pancreatic Fistula**

Grade	A	B	C
Clinical conditions	Well	Often well	Ill, appearing/bad
Specific treatment	No	Yes/no	Yes
Ultrasound/computed tomography (if obtained)	Negative	Negative/positive	Positive
Persistent drainage (after 3 weeks)	No	Usually yes	Yes
Reoperation	No	No	Yes
Death related to POPF	No	No	Possibly yes
Signs of infections	No	No	Yes
Sepsis	No	No	Yes
Readmission	No	Yes/no	Yes/no

Definition of POPF: Output via an operatively placed drain (or a subsequently placed percutaneous drain) of any measurable volume of drain fluid on or after postoperative day 3, with an amylase content >3 times the upper normal serum value (Source: Bassi et al.^1^).

POPF, postoperative pancreatic fistula.

Significant effort has been focused on identifying risk factors that contribute to a POPF, which are generally diagnosed days to weeks after the operation. Veillette et al. used the term occult fistula to refer to patients who initially appear to have no fistula, but who present within weeks to months after an operation with intra-abdominal collections, sepsis, and/or life-threatening hemorrhage.^2^ The occult fistulas in their study were diagnosed no later than 90 days after the operation. We present here a case of a POPF that manifested 8 months after PD. To our knowledge, only one other case has been published of such a late pancreatic fistula, which presented at 7 years.^3^

## Case Presentation

**FIG. 1.**
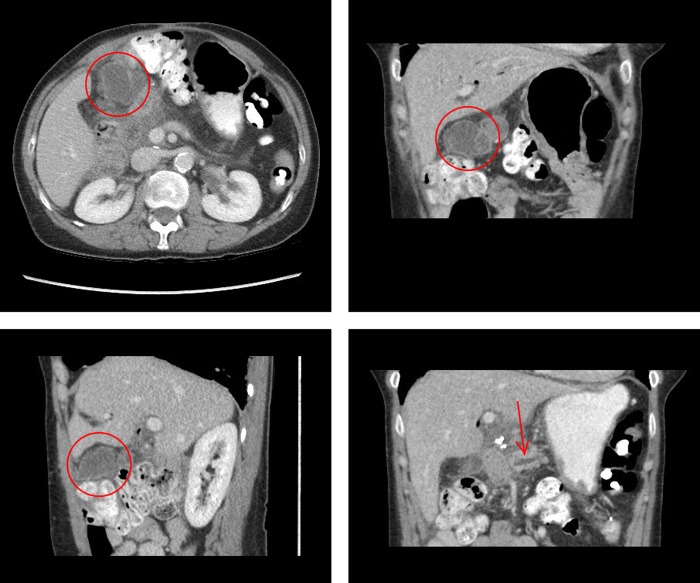
Computed tomography scan identifying a 4.6 × 3 cm collection near the pancreaticojejunostomy (red circle) as well as a prominent proximal pancreatic duct to 4 mm (arrow).

The patient is a 55-year-old female with a body–mass index (BMI) of 23 kg/m^2^, a 20 pack-year smoking history (quit 9 months before presentation), no history of alcohol abuse, and an unremarkable past medical history, who presented for evaluation of newly developed nausea. Her laboratory tests and computed tomography (CT) imaging were all suggestive of biliary obstruction, and she was ultimately diagnosed with an invasive ampullary adenocarcinoma. Staging workup revealed no evidence of metastatic disease, so she was taken to the operating room for a classic PD. A retrocolic hepaticojejunostomy (HJ) was constructed. Similarly, a retrocolic pancreaticojejunostomy (PJ) was created using a duct-to-mucosa technique over a pediatric feeding tube, which was brought out through the lumen of the jejunum and the abdominal wall for external drainage. The pancreatic parenchyma was noted to be very soft, and the pancreatic duct was 2.5 mm in diameter. Estimated blood loss (EBL) was 250 mL. Two 19F Blake drains were left in place above and below the HJ and PJ. Postoperatively, her diet was advanced to soft solids by postoperative day (POD) 4, at which point daily amylase levels of her Blake drainage were checked. One Blake drain was removed on POD5 given an amylase level of 513 U/L. Her remaining Blake drain's amylase level the following day was 6160 U/L, so she was discharged home with plans to remove it in clinic. She was seen on POD13, at which time the drain had an output of <20 mL per day for the past 3 days, so it was removed. She re-presented to our institution on POD23 in transfer from an outside hospital. She endorsed 4 − 5 days of increasing epigastric pain, and a CT of her abdomen and pelvis (CT A/P) demonstrated subphrenic collections, for which she underwent CT-guided placement of two drains. Fluid amylase was 28 U/L and cultures grew *Escherichia coli* and Klebsiella, so she was started on IV antibiotics. Her serum amylase and lipase were 51 and 141 U/L, respectively. One drain was removed by the Interventional Radiology (IR) service 3 days after placement and the second one on follow-up 1 week after discharge, 13 days after placement. She proceeded to adjuvant chemotherapy 7 weeks after her operation and received five cycles of gemcitabine, which she completed successfully. She did not receive any adjuvant radiation. Approximately 8 months after her original operation, she again presented to our Emergency Department with right upper quadrant and epigastric abdominal pain. Her laboratory tests were notable for a white blood cell (WBC) count of 13K/μL and lipase 522 U/L. A CT A/P demonstrated diffuse peripancreatic inflammation. Her pancreatic enzymes rapidly decreased with bowel rest, IV fluid hydration, and supportive care, so endoscopic retrograde cholangiopancreatography (ERCP) was deferred at that time. She was discharged home on hospital day 6 feeling better. Unfortunately, she was again admitted 2 weeks later with similar symptoms. Laboratory tests were notable for a WBC of 17K/μL, amylase 444 U/L, and lipase 677 U/L, and a CT A/P noted a prominent proximal pancreatic duct to 4 mm as well as a 4.6 × 3 cm collection near the PJ (Fig. 1), for which she underwent CT-guided drain placement. The fluid drained had an amylase and lipase of 21,860 and 86,650 U/L, respectively, and cultures were sterile, findings consistent with a pancreatic fistula.

**FIG. 2.**
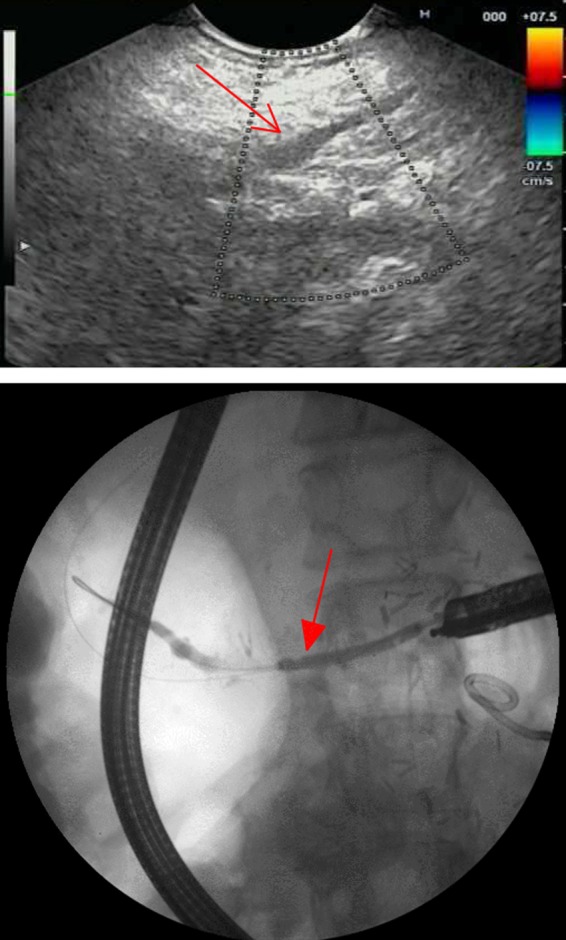
Top—EUS image highlighting a dilated pancreatic duct (simple arrow). Bottom—Fluoroscopy image demonstrating adequate placement of a 7F 9-cm Hobbs stent with internal flange across the newly dilated pancreaticojejunostomy (filled-head arrow). EUS, endoscopic ultrasound.

She underwent an ERCP and attempt at pancreatic duct cannulation, which was unsuccessful due to the inability to identify the latter from within the jejunum. Four days later, she underwent a repeat endoscopy and a complex rendezvous procedure was performed. The pancreatic duct was identified from within the stomach using endoscopic ultrasound (EUS), with subsequent transgastric transpancreatic passage of a wire into the duct and out to the jejunum. This was followed by ERCP and rendezvous identification of the wire, dilation of the PJ using a 4-mm Titan balloon, and placement of a 7F 9-cm Hobbs stent with an internal flange at the PJ with good drainage^4^ (Fig. 2). Her symptoms rapidly improved, and she was discharged home on hospital day 8.

## Review of Literature and Discussion

We have described the case of a woman with a POPF diagnosed 8 months after PD for invasive ampullary adenocarcinoma. To our knowledge, this is the second latest POPF published in the current literature.^3^ Pancreatic fistulas after PD are a dreaded complication, one that occurs not infrequently and that confers a statistically and clinically significant increase in morbidity and mortality. The rates of POPF formation after PD have been reported to be 9% to 17% (average 13%),^2,5–12^ but as high as 24%,^13^ leading to a two- to threefold increase in hospital length of stay (26 days vs. 13.2 days,^5^ 23.6 days vs. 8.7 days,^2^ 25 days vs. 12 days^8^) and rates of readmission 1.5 to 2.5 times higher (26.7% vs. 11.1%,^2^ 17.2 vs. 11.3^9^) when compared with patients who do not develop this complication. Rates of reoperation (7.9–14% vs. 0.42–2.7%)^5,8,14^ and IR drainage (37.3% vs. 4.7%)^2^ are significantly higher as well. Mortality rates as high as 28%^15^ have been reported. Veillette et al. found an almost eightfold increase in mortality for patients who develop a POPF (9.3% vs. 1.2%), with POPF-related deaths noted as far out as 92 days after the operation.^2^ More recently, Addeo et al. reported on a cohort of 1325 patients at 37 institutions and similarly found a nearly sixfold higher risk of 60-day in-hospital mortality (13.3% vs. 2.3%)^9^ for patients who develop a POPF.

The search for risk factors for POPF has been extensive and ongoing for many decades. Pertaining to tumor anatomy and biology, PDs performed for pancreatic adenocarcinoma have been reported to have a lower risk of POPF formation than those performed to resect ampullary,^2,6–8,15^ duodenal,^6–8^ distal bile duct,^7^ cystic, or islet cell^6^ malignancies. Two of the most generally accepted risk factors for the formation of POPF are pancreatic parenchymal texture and pancreatic duct diameter. POPFs are 4 to 18.6 times more likely to occur among patients with a soft or normal pancreatic parenchyma than in those with a fibrotic one.^5,6,9,14^ Similarly, patients who develop a POPF have been found to be 2.5 to 3 times more likely to have a pancreatic duct diameter of <2–3 mm.^6,15,16^ The strong relationship between pancreatic duct size and parenchyma texture with the development of a POPF is thought to be related to the difficulty of constructing the anastomosis as well as the likelihood of the pancreatic tissue to hold sutures.

Factors associated with the surgeon and surgical technique include the type of anastomosis made and the EBL of the operation. Variable results have been presented regarding type of anastomosis, and several different techniques have been proposed. First off, one must decide whether a pancreaticogastrostomy (PG) versus PJ should be constructed. Three separate randomized controlled trials (RCTs) were conducted to explore the differences in outcomes between PG and PJ, and all three were unsuccessful in elucidating any difference.^17–19^ Nevertheless, a more recent RCT by Topal et al. in 2013 found that patients with a pancreatic duct <3 mm in diameter who underwent reconstruction via PJ had an almost threefold higher risk of POPF formation than those with reconstruction via PG, but this effect did not transfer to those patients with a pancreatic duct diameter of >3 mm.^20^ Different techniques to construct a PJ have similarly been described, including end-to-side single layer, end-to-side duct-to-mucosa, end-to-end invaginated, and end-to-end binding invaginated anastomoses. Bassi et al. in 2003 compared the end-to-side single-layer versus duct-to-mucosa techniques and found no difference in the rate of POPF formation.^21^ Schmidt et al. compared the duct-to-mucosa versus the end-to-end invaginated anastomoses and found the latter to be an independent risk factor for the development of POPF, conferring a 4.5-fold increased risk. Furthermore, they analyzed the subpopulation of patients who had periampullary tumors and noted an even more impressive association, with invagination leading to almost 12 times higher odds of developing a POPF.^8^ Peng et al. published their end-to-end binding invaginated technique for construction of a PJ and presented a 0% rate of POPF formation,^22^ but other studies have been unable to replicate these results. Finally, Callery et al. in 2013 determined that EBL >1000 mL was associated with a 5.6-fold higher likelihood of developing a POPF,^6^ a finding that has been supported by several other studies.^5,23^ This is thought to result from the ensuing ischemic insult to the fragile cut edges at the anastomotic site.

Several groups have attempted to develop predictive models that estimate the likelihood of developing a POPF. Roberts et al. in 2013 developed a preoperative risk prediction model with an area under the curve (AUC) of 0.832 using all retrospective data, which relies on two factors: BMI and pancreatic duct diameter. Utilizing our patient's BMI of 23 kg/m^2^ and pancreatic duct diameter of 2.5 mm (although this is a postop value in our case), their model predicts a 28% risk of developing a POPF. Similarly in 2013, Callery et al. analyzed retrospective data from 233 patients and constructed a model with an AUC of 0.942, utilizing four factors validated in a prospective manner: pancreatic parenchyma texture, ampullary malignancy or not, pancreatic duct diameter, and EBL.^6^ Using their model, we would have predicted a 13.8% to 17.6%^24^ risk of developing a clinically relevant (group B or C ISGPF) POPF. More recently, in 2016, Sato et al. published their study of 87 consecutive patients undergoing PD and aimed at developing a simpler binary predictor of the likelihood of developing a POPF versus not. They analyzed data retrospectively and created a model with an AUC of 0.781, which uses the ratio of BMI to prognostic nutritional index (PNI, calculated as 10 × serum albumin (g/dL) +0.005 × total lymphocyte count per mm^3^) >0.5 to predict the development of POPF.^12^ Using their model and our patient's preoperative values (BMI 23 kg/m^2^, albumin 3.3 g/dL, and total lymphocyte count 1770/mm^3^), we obtain a BMI/PNI of 0.55, which would have accurately predicted that our patient would develop a POPF.

In a patient with a soft pancreatic gland, a small pancreatic duct diameter (2.5 mm), and a diagnosis of ampullary adenocarcinoma, a POPF is not unexpected. What is worthy of note is the timing at which her POPF manifested, that is, 8 months after her operation. We hypothesize that the severe stenosis at the PJ, which was identified on CT A/P and confirmed by ERCP, led to leakage of pancreatic enzymes and a delayed POPF. Four studies have been published exploring the rate of PJ stricture (PJS) formation after PD. Reid-Lombardo et al. reported on 122 patients undergoing PD and identified a PJS rate of 3.3%, with 1- and 5-year cumulative probabilities of development of 2.8% and 4.6%, respectively. They did not seek to identify risk factors.^25^ Callery and colleagues in 2010 found the incidence of PJS formation to be 2% (7/357 patients) and identified the prior formation of a POPF (6/7 patients) as a contributing factor.^26^ Morgan et al. reported an 11.3% incidence of PJS in their cohort of 237 patients, most of whom underwent PD for chronic pancreatitis (68%), and indeed identified chronic pancreatitis as a risk factor for PJS (*p* < 0.04). Interestingly, 14.8% of the patients who developed PJS also developed pancreatic pseudocysts.^27^ More recently, in 2016, Cioffi et al. reported a PJS rate of 2.2% in their cohort of 1175 patients, for whom the vast majority of PDs were performed for pancreatic adenocarcinoma. They were unable to identify significant risk factors, but did note a trend toward higher rates of PJS formation in patients who received adjuvant chemoradiation. The median time from the operation to diagnosis was 46 months (range 3.5–270 months). Acute pancreatitis and abdominal pain were the two ways in which patients presented at the time of diagnosis.^28^

In reviewing our patient's presentation, her significant smoking history stands out as a potential risk factor for PJS formation. Cioffi et al. specifically examined this question in their study and were unable to identify a relationship. Nevertheless, smoking is known to create a thrombogenic environment in the human vasculature, vasoconstriction, and failure of oxygen delivery^29^ and has been found to be a strong risk factor for the development of anastomotic strictures in other sites such as the esophagogastric anastomosis after Ivor Lewis esophagectomies for esophageal cancer,^30^ the colorectal anastomosis after low anterior resections for rectal cancer,^31^ and perhaps more similar to our case, at the biliary anastomosis after liver transplant surgery.^29^ Given the paucity of data on PJS after PD, one can propose that a relationship similar to that ascertained in the aforementioned studies remains to be elucidated.

In synthesizing the aforementioned data, we can hypothesize that our patient may have incurred a higher risk of PJS formation secondary to her adjuvant chemotherapy as well as her history of POPF, which manifested initially as pancreatitis and phlegmonous changes on CT A/P. She then may have developed a leakage of pancreatic enzymes, which eventually led to her delayed POPF. By performing an EUS and rendezvous ERCP to place a PJ stent, her pancreatic remnant was adequately decompressed and her symptoms subsided.

## Conclusion

POPFs remain a significant complication after PD. Most POPFs occur days to weeks after a pancreatic resection, but can extend out as far as several months postoperatively. A clear relationship between POPFs and PJS has not been elucidated, but it is reasonable to presume an interplay between the two. In patients presenting with symptoms of recurrent pancreatitis after PD, the PJ should be evaluated. If a stricture is identified, prompt decompression of the pancreatic remnant should be performed, which may in turn prevent worse complications and provide patients with effective and rapid resolution of their symptoms.

## Abbreviations Used

AUC: area under the curve
BMI: body–mass index
CT A/P: computed tomography of abdomen and pelvis
EBL: estimated blood loss
ERCP: endoscopic retrograde cholangiopancreatography
EUS: endoscopic ultrasound
HJ: hepaticojejunostomy
IR: interventional radiology
ISGPF: International Study Group on Pancreatic Fistula
PD: pancreaticoduodenectomy
PG: pancreaticogastrostomy
PJ: pancreaticojejunostomy
PJS: PJ stricture
PNI: prognostic nutritional index
POD: postoperative day
POPF: postoperative pancreatic fistula
RCT: randomized controlled trials
WBC: white blood cell
